# Barbed sutures versus conventional sutures for wound closure in spine surgeries: a systematic review and meta-analysis

**DOI:** 10.1007/s10143-024-02909-9

**Published:** 2024-10-10

**Authors:** Khalid Sarhan, Reem Reda Elmahdi, Rashad G. Mohamed, Ibrahim Serag, Mohamed Abouzid

**Affiliations:** 1 Faculty of Medicine, Mansoura University, Mansoura, Egypt; 2 Mansoura Manchester Program for Medical Education, Faculty of Medicine, Mansoura University, Mansoura, Egypt; 3 Department of Physical Pharmacy and Pharmacokinetics, Faculty of Pharmacy, Poznan University of Medical Sciences, Rokietnicka 3 St., Poznan, 60-806 Poland; 4 Doctoral School, Poznan University of Medical Sciences, Poznan, 60-812 Poland; 5 Medical Research Group of Egypt (MRGE), Negida Academy, Arlington, MA USA

**Keywords:** Barbed sutures, Conventional sutures, Spinal surgery, Wound closure, Wound complications, Meta-analysis

## Abstract

Suturing techniques for wound closure in spine surgeries play a critical role in patient outcomes, including wound healing, reintervention, and risk of complications. Barbed sutures, characterized by their self-anchoring properties, have emerged as a potential alternative to conventional sutures in various surgical disciplines. While previous studies have underscored their efficacy and safety in spine surgeries, no meta-analysis has been conducted. Therefore, we are undertaking this study. Following the PRISMA guidelines, we conducted a literature search on electronic databases to obtain the relevant studies until May 5, 2024. Our primary outcomes were operative time, wound closure time, and postoperative wound complications like seroma or hematoma formation and wound infection. The secondary outcomes were the length of hospital stay, reintervention rates, and costs. Data was pooled using a random effects model. We included seven eligible studies with a total of 8645 patients. Our meta-analysis showed that barbed sutures had shorter operative time and wound closure time compared to conventional sutures (MD -20.13 min, 95% CI [-28.47, -11.78], *P* < 0.001) and (MD -16.36 min, 95% CI [-20.9, -11.82], *P* < 0.001), respectively. Both suturing techniques showed comparable results in terms of overall postoperative wound complications (RR 0.83, 95% CI [0.60, 1.14], *P* = 0.25), postoperative infections (RR 0.59, 95% CI [0.33, 1.06], *P* = 0.08), length of hospital stay (MD -0.26 day, 95% CI [-0.75, 0.22], *P* = 0.28), rates of reintervention between the two groups (RR 0.99, 95% CI [0.48, 2.05], *P* = 0.98). Barbed sutures in spine surgeries are associated with significantly shortened wound closure and operative times. However, high-quality RCT’s with long-term follow-up and cost-effectiveness assessment are required to support the evidence.

**Supplementary Information:**

The online version contains supplementary material available at 10.1007/s10143-024-02909-9.

## Introduction

Spine-related disorders are among the most common causes of years lived with disability according to the Global Burden of Disease study [1]. In recent decades, these disorders have benefited from substantial advancements in rehabilitation practices, prehospital care, diagnostic methodologies, therapeutic interventions, and surgical technologies [2, 3]. Complications after spine surgery pose a significant concern due to their impact on increased morbidity, mortality, longer hospital stays, and higher healthcare costs [4]. The rate of complications in spinal surgeries is estimated to range from 13 to 40%. Among these, postoperative wound complications are among the most frequently reported after spine procedures [5]. Successful wound closure, an essential part of accelerated recovery after surgery, is crucial to attaining positive postoperative outcomes after spine surgery, as it may have an impact on healing, the risk of surgical site infection, post-acute care follow-up, and the need for surgical correction of the wound [6, 7]. The conventional method of closing surgical wounds using knotted suture materials has several limitations that pose significant challenges, such as increased risk of infections, scarring, and hematoma formation [8].

**Fig. 1:**
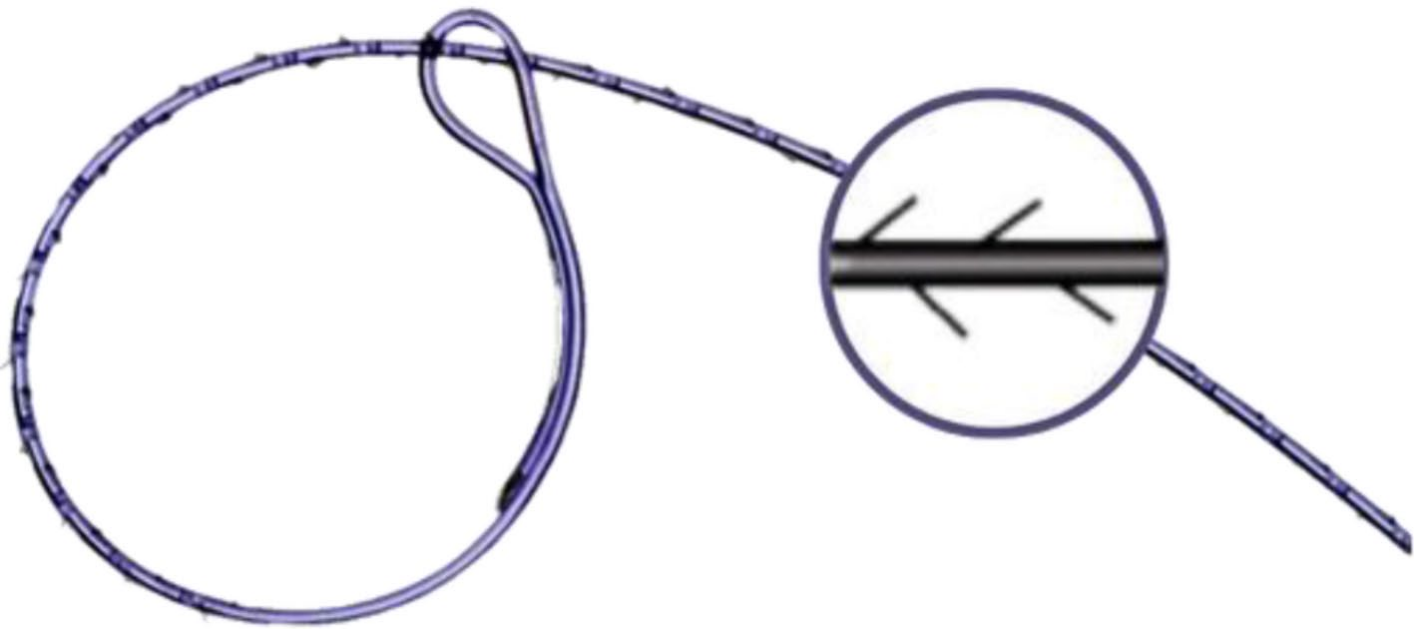
This figure shows the knotless unidirectional barbed suture illustrating its densely arranged unidirectional barbs that have the advantage of maintaining tension owing to the self-locking and multi anchor properties

Barbed sutures, knotless or self-retaining sutures, are absorbable sutures that integrate several spikes (barbs) on their outer surface Fig. 1. These spikes can penetrate tissues without the need to tie knots, which saves considerable time during suturing [9]. Nowadays, barbed sutures address certain drawbacks associated with conventional sutures, as they are often associated with shorter suturing times, quicker wound closure, cheaper hospital expenses, and fewer postoperative problems than traditional ones [10–12]. Recently, several studies have been conducted to evaluate the use of barbed sutures in different surgical fields like knee arthroplasty, obstetrics, gynecology, cosmetology, and tenorrhaphy [13–15]. These studies revealed that barbed sutures may overcome traditional sutures by providing greater wound strength, minimizing complications, and saving time and costs. Although some studies [16–18] evaluated the differences between conventional sutures with knots and knotless barbed sutures, scant evidence exists regarding the efficacy of barbed sutures in spinal surgeries, particularly concerning clinical outcomes, complications, and suture costs. Therefore, our systematic review and meta-analysis aimed to compare the outcomes of barbed sutures versus conventional suturing techniques for wound closure following spine surgery.

## Materials & methods

### Protocol

This systematic review and meta-analysis were carried out following the established protocols provided by PRISMA (Preferred Reporting Items for Systematic Reviews and Meta-Analyses) [19]. Our protocol was registered on PROSPERO (CRD42024573883) and OSF registries 10.17605/OSF.IO/HDA6S. The retrieval of the study, eligibility screening, data extraction, and quality assessment were completed independently by each author.

### Search strategy

We conducted a comprehensive search until May 5, 2024, across four databases: PubMed, Scopus, Web of Science, and the Cochrane Library, looking for relevant studies. Geographical or publication-status restrictions were not imposed. The search approach used MeSH (medical subject headings) and their synonyms. “Back and Spine Surgery”, “wound closure”, “barbed sutures”, “knotless sutures”, “traditional sutures” and “conventional sutures” were the search terms utilized. Each database’s requirements dictated modifications to the vocabulary and syntax, and the Boolean operators “AND” and “OR” were used to combine the terms. Furthermore, the reference lists of pertinent articles were thoroughly examined to ensure no items were possibly overlooked.

### Eligibility criteria

This analysis only included clinical studies that satisfied the specified criteria: [1] Any randomized or non-randomized control trials; and cohort studies (prospective or retrospective) [4] studies with adult patients who underwent elective posterior spine surgery in any region of the spine; [5] post-surgical wound closure using the traditional closure technique or the barbed knotless suture closure technique; [6] operation time, duration of wound closure, postoperative wound complications, incision size, hospitalization time, blood loss, and costs as outcomes. Studies were excluded if they were: [1] non-English; [4] other study designs like case reports, case series, nonmedical papers, reviews, conference abstracts, animal or in vitro studies, and pre-clinical studies; [5] studies involving other techniques for wound closure, e.g. (skin adhesives or other suturing techniques); [6] studies including surgeries anywhere else aside from the spine were also excluded.

### Outcome definition

Our primary outcomes were operative time, wound closure time, and postoperative wound complications like seroma or hematoma formation and wound infection. The secondary outcomes were the length of hospital stay, reintervention rates, and costs.

### Study selection

Two researchers (RGM and RRE) independently examined the title and abstract of each study to remove any unnecessary studies after eliminating duplicates using Endnote X9. After that, we screened the complete texts of the studies that satisfied our preset inclusion criteria. Any differences were resolved by consensus-building with a third reviewer.

### Quality assessment and publication bias

The Cochrane risk-of-bias assessment tool (ROB-2) was used to evaluate the quality of the only randomized controlled trial included [20]. The Newcastle-Ottawa Quality Assessment Scale (NOS), which is based on three general parameters: group comparability, exposure or outcome of interest ascertained, study group selection, and representativeness, was employed by two independent researchers (RGM and RRE) to assess the quality of the included cohort studies [21]. Based on the following score ranges, each research quality was divided into three groups: 0 to 3, 4 to 6, and 7 to 9. These ranges represented three study quality levels: good, fair, and poor, respectively. If the study took 0 in the comparability domain, the overall quality of the study was judged as poor quality. Publication bias was evaluated using a funnel plot with ten or more included papers [22].

### Statistical analysis

Risk ratio (RR) and 95% confidence interval (CI) were computed for dichotomous variables (e.g., total number of postoperative wound complications, frequency of dehiscence or seroma formation, hematoma, postoperative wound infection, and frequency of reinterventions due to wound healing problems). For continuous variables (e.g., operation time, wound closure time, and length of hospital stay), the mean difference (MD) and 95% CI were computed to pool effect estimates. Any result presented as the median and interquartile range (IQR) was converted to mean and standard deviation (SD) using the Wan et al. [23] technique. When a study reported mean and 95% CI, standard deviation was hypothetically determined to match the reported 95% CI by the original study. Because the heterogeneity between studies was predicted, we employed a random-effects model. Heterogeneity was measured using the Higgins I² test, with 0–40% regarded as minor, 30–60% as moderate, 50–90% as substantial, and 75–100% as considerable heterogeneity [24]. Sensitivity analyses, where we remove studies one at a time, were used to see how robust the results were and to gauge each study’s contribution. We used Review Manager V.5.3 and Open Meta-Analyst software ^®^ for our statistical analysis.

## Results

### Study selection

**Fig. 2:**
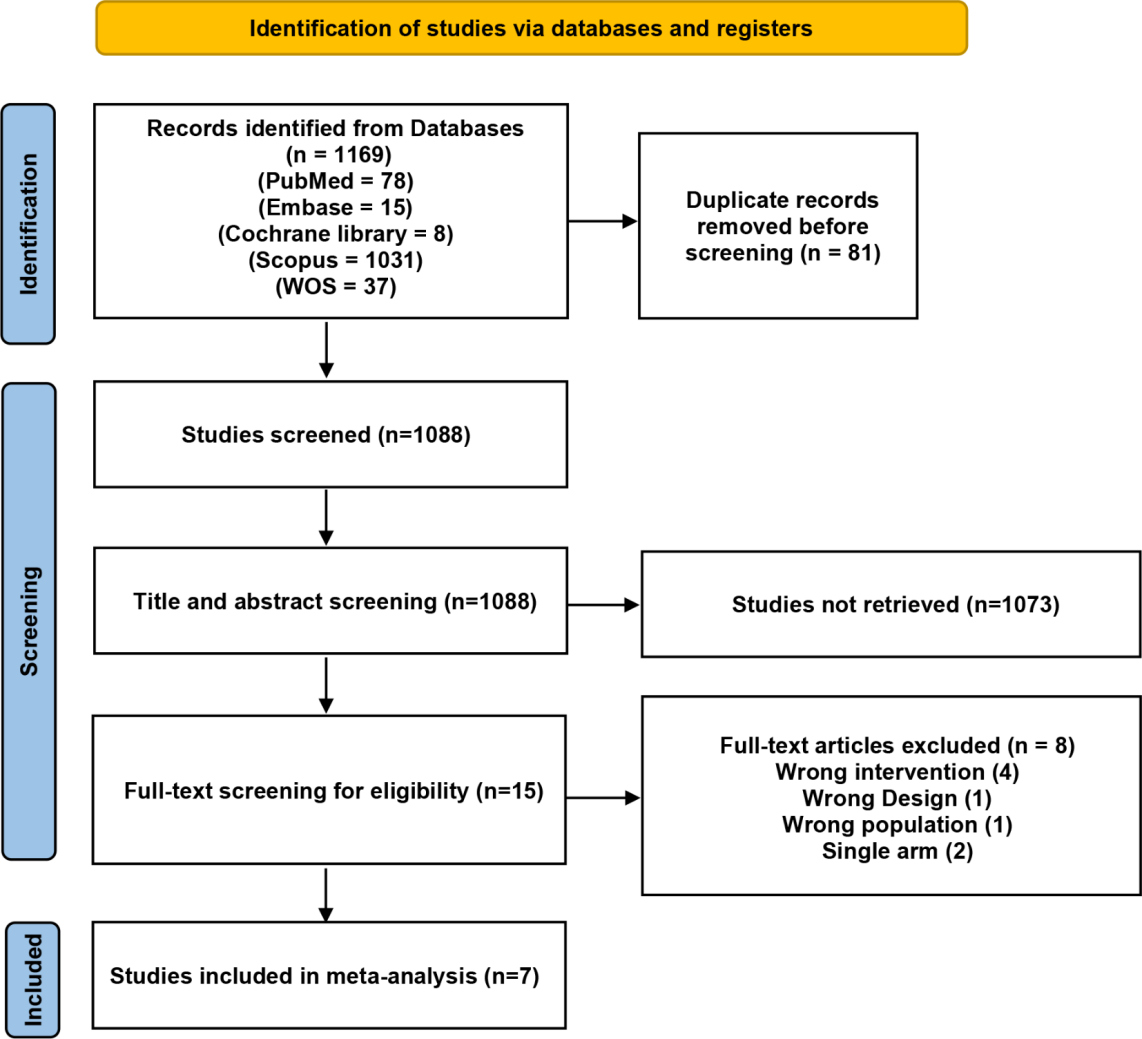
Prisma flow diagram of the included studies

Our search retrieved 1154 studies. Following title and abstract screening, 15 studies were eligible for full-text screening. A total of 7 studies with 8645 patients met the eligibility criteria and were included in this meta-analysis (Fig. 2). Of the seven studies comparing barbed sutures and conventional sutures in patients undergoing spine surgeries, barbed sutures were used in 4240 patients, while conventional sutures were used in 4405 patients. A summary of the included studies is presented in Table 1.

**Table 1:** Baseline characteristics of the included studies

Study ID	Study Design	Study Funding	Country	Sample size		Age (Mean, SD)		Sex (F) (*N*%)		Follow-up period (months)
				BS	CS	BS	CS	BS	CS	
Chen, 2018 [8]	Retrospective Cohort	Funded by the National Natural Science Foundation of China and Wenzhou Science and Technology Project	China	98	91	50.43 (9.56)	50.18 (10.06)	28 (29%)	68 (75%)	15
Delgado-López, 2024 [25]	Retrospective Cohort	No funding	Spain	183	300	59.25 (13.49)	57.75 (13.09)	78 (43%)	119 (40%)	10
Johnston, 2020 [26]	Retrospective Cohort	Funded by Johnson and Johnson	USA	3705	3705	61.6 (12.9)	61.3 (12.9)	2021 (55%)	2021 (55%)	NA
Mansour, 2013 [27]	Prospective Cohort	Not reported	USA	15	10	15.4 (3.2)	13.0 (2.3)	11 (73%)	7 (70%)	NA
Mun, 2023 [28]	Retrospective Cohort	Funded by Johnson and Johnson	Korea	120	120	56.7 (20.7)	57.9 (20.4)	71 (59%)	71 (59%)	NA
Shi, 2022 [29]	RCT	No funding	China	91	90	55.85 (17.1)	52.97 (19.92)	35 (38%)	55 (61%)	NA
Tang, 2022 [30]	Retrospective Cohort	No funding	USA	28	89	63.4 (12.5)	61.3 (12.8)	15 (54%)	56 (63%)	3

BS: barbed sutures, CS: conventional sutures, F: female, SD: standard deviation

### Baseline characteristics

Overall, the average age of the patients was 51 years, and it ranged from 13 to 63 years, and 4656 (54%) were females. Two studies were conducted in the USA, two in China, one in Spain, and one in Korea. The baseline characteristics of the included studies are presented in Table 1. The suturing type and placement method for the fascia, subcutaneous, intradermal, and skin layers in both barbed and conventional sutures in all included studies are presented in Table 2.

**Table 2:** Details of suture type and method of placement in spine surgeries

Study ID	Barbed sutures					Traditional sutures			
	Fascia	Subcutaneous	Intradermal	Skin	Fascia		Subcutaneous	Intradermal	Skin
Chen, 2018 [8]	Bidirectional size 2 spiral PDO Stratafix	NA	Bidirectional size 2 spiral PDO Stratafix	Bidirectional size 2 − 0 spiral PGA-PCL Stratafix	Interrupted size 0 Vicryl		NA	Interrupted 2 − 0 Vicryl	Interrupted 4 − 0 Vicryl
Delgado-López, 2024 [25]	1/0 or 0/0 running barbed sutures Stratafix	1/0 or 0/0 running barbed sutures Stratafix	NA	Running 4/0 non-absorbable monofilament or staples	2/0 absorbable monofilament or 1/0 monofilament		3/0 Monocryl	NA	NA
Johnston, 2020 [26]	Unclear	Unclear	Unclear	Unclear	Unclear		Unclear	Unclear	Unclear
Mansour, 2013 [27]	Bidirectional closure: interrupted absorbable sutures in a figure of eight manner + Running size 2 PDO; Quill SRS, Angiotech	NA	Single running size 0 PDO; Quill SRS, Angiotech	Running 3 − 0 Monoderm; Quill SRS, Angiotech	Interrupted absorbable suture in a figure of eight manner size 1 Vicryl		NA	Inverted simple interrupted 2 − 0 Vicryl	Running absorbable 3 − 0 Monocryl
Mun, 2023 [28]	Unidirectional barbed sutures	Bidirectional barbed sutures	NA	Self-adhering mesh and polymeric glue	Vicryl interrupted sutures		Vicryl interrupted sutures	NA	Staples
Shi, 2022 [29]	Continuous size 2 Fenkuill sutures	NA	Continuous size 2 or size 1 Fenkuill sutures	Continuous size 2 − 0 Fenkuill sutures	Interrupted absorbable suture size 0 Vicryl		NA	2 − 0 Vicryl sutures	3 − 0 Vicryl sutures
Tang, 2022 [30]	#1 Stratafix running barbed suture	NA	2 − 0 Vicryl suture	2 − 0 Nylon sutures	Interrupted #1 Vicryl sutures in a figure of eight fashion		NA	2 − 0 Vicryl sutures	2 − 0 Nylon sutures

### Risk-of-bias assessment

**Fig. 3:**
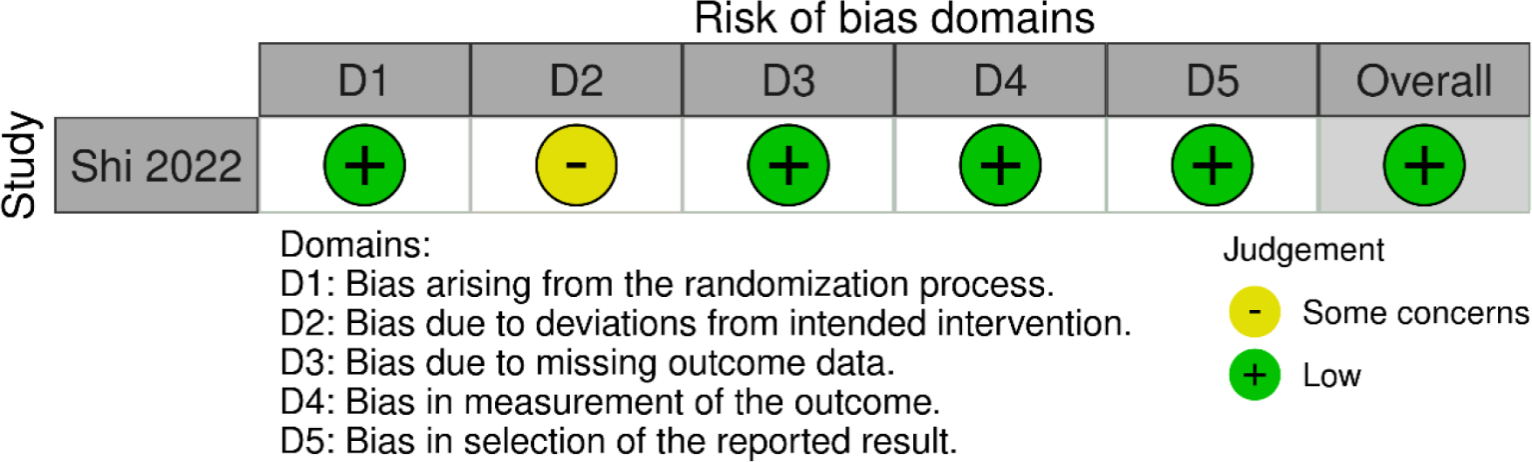
Risk-of-bias assessment using ROB-2 for the only randomized controlled trial included

One RCT and six comparative cohort studies were included in this study. We used the ROB-2 tool to assess the risk-of-bias for the included RCT, with the following findings: Randomization process: low risk; deviations from the intended interventions: some concerns; bias in the measurement of the outcome: low risk; bias due to missing outcome data: low risk; bias in the selection of the reported results: low risk; other bias: low risk. The overall assessment was low risk Fig. 3. We used the NOS scale for the remaining six cohort studies, one of which was deemed poor quality, and the rest were good quality. A summary of the risk-of-bias assessment is presented in Table 3.

**Table 3:** Risk-of-bias assessment using NOS

Study ID	Selection	Comparability	Outcome	Overall	Final decision
Chen 2018 [8]	4	1	3	7	Good quality
Delgado-López 2024 [25]	3	0	3	6	Poor quality
Johnston 2020 [26]	3	2	3	8	Good quality
Mansour 2013 [27]	4	1	2	6	Good quality
Mun 2023 [28]	4	1	2	7	Good quality
Tang 2022 [30]	4	2	3	9	Good quality

### Results of the primary outcomes

#### Operative time (minutes)

**Fig. 4:**
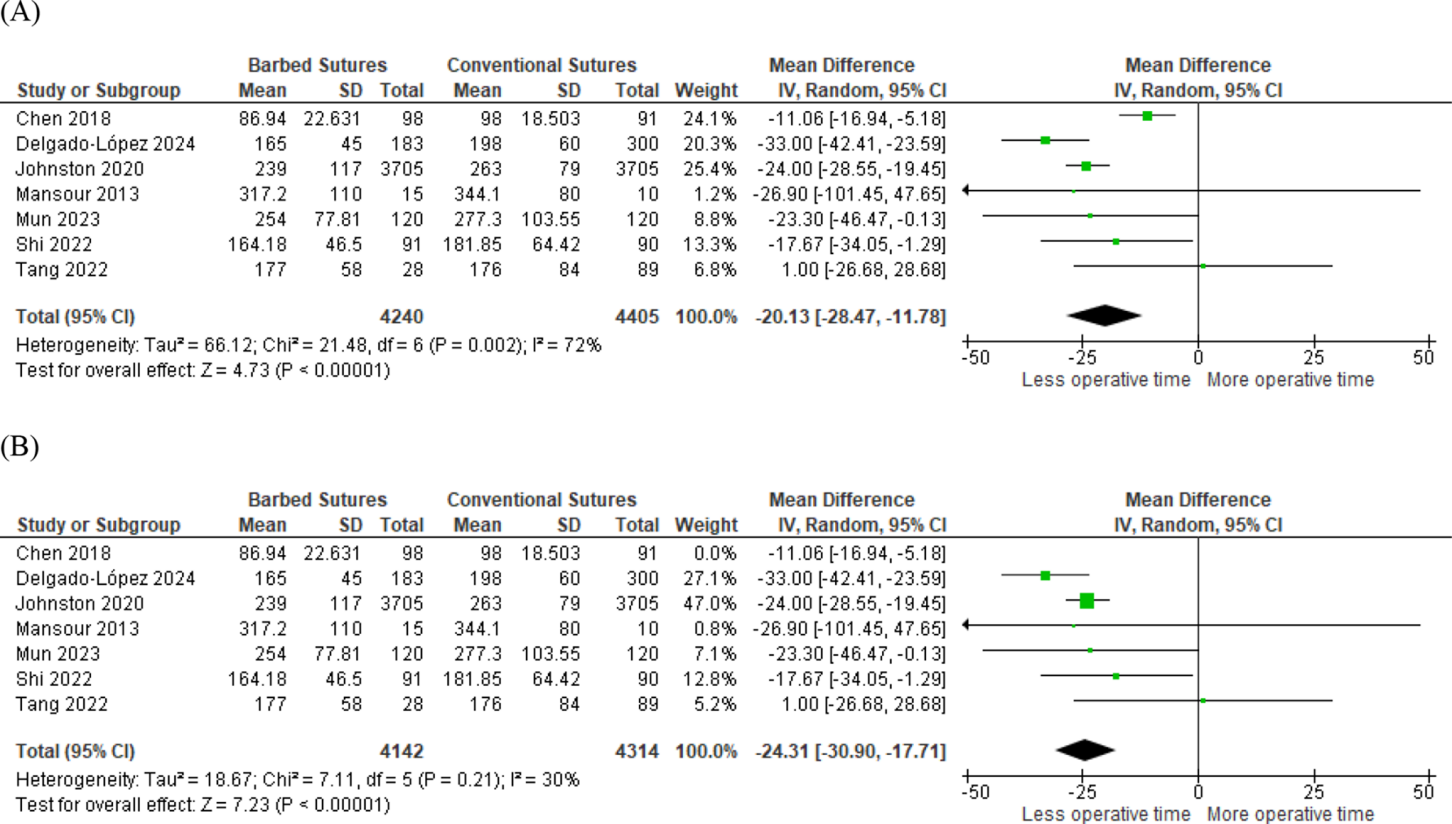
Forest plots of operative time (**A**) before sensitivity analysis (**B**) after excluding Chen et al. study

Operative time was reported in all seven studies, including 8645 patients. We found that using barbed sutures significantly reduced the actual operation time compared to conventional sutures (MD -20.13, 95% CI [-28.47, -11.78], *P* < 0.001). Significant heterogeneity was found between the effect sizes of the included studies (*P* < 0.001, I² =82%) Fig. 4a. We conducted a sensitivity analysis in multiple scenarios, excluding one study, to resolve this heterogeneity. Heterogeneity was best determined by excluding the study of Chen et al. [8] (*P* = 0.21, I² = 30%). After removing this study from the meta-analysis model, the overall mean difference was still in favor of barbed sutures (MD -24.31, 95% CI [-30.9, -17.72], *P* < 0.001) Fig. 4b.

#### Wound closure time (minutes)

**Fig. 5:**
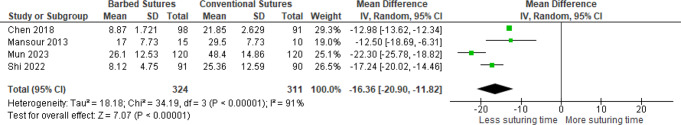
Forest plot of wound closure time

Four studies, including 635 patients, compared the suturing time between barbed and conventional sutures. Suturing time was significantly reduced in the barbed sutures group compared to conventional sutures (MD -16.36, 95% CI [-20.9, -11.82], *P* < 0.001). Substantial heterogeneity between the studies could not be resolved by sensitivity analysis (*P* < 0.0001, I² = 91%) Fig. 5.

#### Postoperative wound complications

**Fig. 6:**
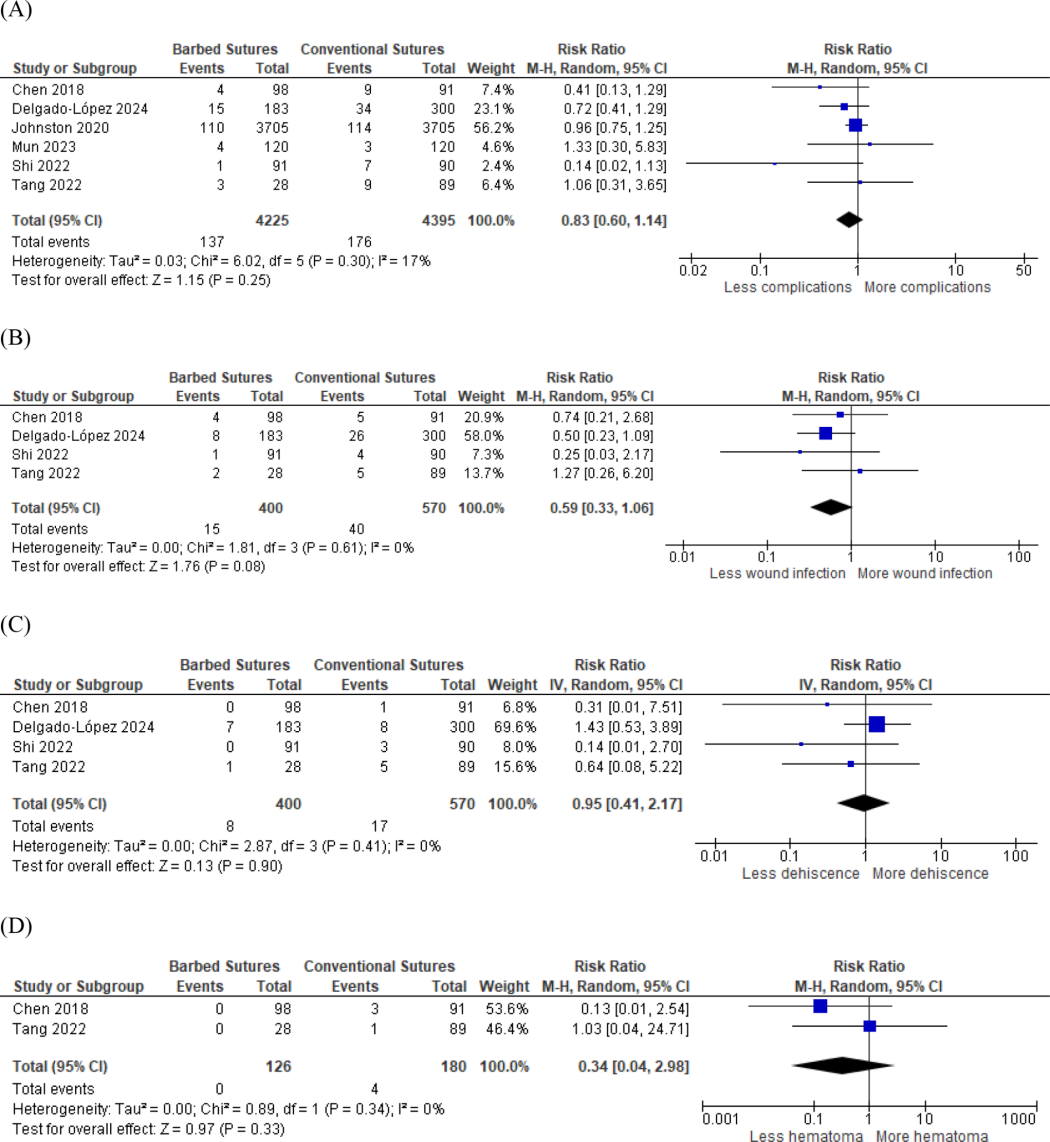
Forest plots of post-operative wound complications (**A**) overall post-operative wound complications (**B**) wound infections (**C**) dehiscence or seroma (**D**) hematoma

The overall postoperative wound complications were assessed in six studies, including 8620 patients. Barbed and conventional sutures did not differ statistically significantly (RR 0.83, 95% CI [0.60, 1.14], *P* = 0.25). No heterogeneity was found between the effect sizes (*P* = 0.3, I² = 17%) Fig. 6a. Four studies assessed postoperative wound infection and dehiscence or seroma, including 970 patients. Lower rates of wound infection were found in the barbed sutures group. However, the overall risk ratio was not statistically significant compared to conventional sutures (RR 0.59, 95% CI [0.33, 1.06], *P* = 0.08) Fig. 6b. No significant difference was found between the two groups regarding wound dehiscence or seroma (RR 0.95, 95% CI [0.41, 2.17], *P* = 0.90) Fig. 6c. No heterogeneity was found in wound infection outcome (*P* = 0.61, I² = 0%) and dehiscence or seroma (*P* = 0.41, I² = 0%). Two studies assessed the rates of postoperative hematoma. In the barbed sutures group no reports of hematoma formation were found in both studies. However, in the conventional sutures group 3/91 patients and 1/89 patients developed postoperative hematoma in Chen 2018 and Tang 2022 respectively Fig. 6d.

### Results of the secondary outcomes

#### Length of hospital stay (days)

**Fig. 7:**
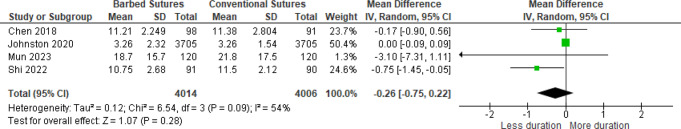
Forest plot of length of hospital stay following surgery

Length of hospital stay was assessed in four studies, 8020 patients. There was no difference between barbed sutures and conventional sutures (MD -0.26, 95% CI [-0.75, 0.22], *P* = 0.28). However, substantial heterogeneity between the studies (*P* = 0.09, I² = 54%) Fig. 7. It could not be resolved by sensitivity analysis.

#### Reintervention

**Fig. 8:**
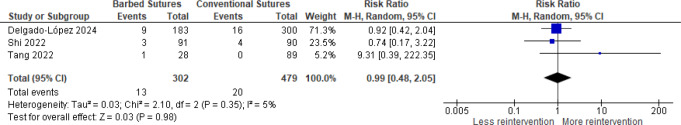
Forest plot of reintervention rates

Three studies assessed the rates of reintervention between the two groups. There was no difference between barbed and conventional sutures (RR 0.99, 95% CI [0.48, 2.05], *P* = 0.98). No heterogeneity was found (*P* = 0.35, I² = 5%) Fig. 8.

## Discussion

We found that using barbed sutures significantly reduced the actual operation time and suturing time compared to conventional sutures. No statistically significant difference was found between barbed and conventional sutures regarding shorter hospital stay duration, postoperative wound complications, or reintervention.

A single barbed suture is used to secure the muscle-fascial and subcutaneous layers. In conventional sutures, at least three layers (muscle, fascia, and subcutaneous) must be closed independently before subcuticular and epidermal sutures are performed [25]. While this method is widely recognized and practiced, it presents inherent challenges, particularly regarding the strength of wound closure and the time required. Wounds resulting from revision surgeries often involve scar tissue, necessitating more tension than normal skin [26–28]. Consequently, suturing in revision surgeries demands a longer closure duration. The first patent for barbed sutures was granted in 1964 [29]. The initial design of these sutures was unidirectional, which restricted their use as the surgeon had to “double back” to secure the closure [29]. Over time, the design and application of barbed sutures have evolved significantly. The first study on using barbed sutures in spinal surgeries was conducted by Mansour et al. [30] as a quality improvement project. It highlighted the economic and operational advantages of using barbed sutures in spinal surgeries. Authors reported that using barbed sutures to close spinal fusion incisions has been shown to reduce closure time by 40%, leading to a decrease in hospital charges related to operating room time by $884.60. This reduction in time and cost is particularly significant in high-volume spine fusion centers and provides a preliminary economic justification for adopting barbed sutures [30]. In our analysis, suturing time was significantly lower by approximately 16 min when barbed sutures were used.

Regarding the actual operation time, it may be evident that a lower suturing time may lower the overall operating time. Hence, the findings were consistent among all studies except in Mansour 2013 [30] and Tang 2022 [31], as these studies had the lowest number of patients involved, with 15 and 28 patients, respectively. Hence, statistical power was not achieved, and both had a higher confidence interval. Therefore, the operating time would be less than 24 min if barbed sutures were used. It is essential to note that the time required for wound closure may increase directly to the number of operated segments when employing the interrupted suture technique which may have contributed to the presence of heterogeneity in our results [26–28].

Despite the shorter wound closure and actual operation time, there were no differences in length of hospital, reintervention rates, and postoperative wound complications, including overall postoperative wound complications, wound infections, dehiscence or seroma, and hematoma. Notably, several papers highlighted the mechanisms by which barbed sutures might reduce wound infection rates. Delgado-López [25] underscored the superior physical properties of barbed sutures (barbed sutures), which include self-tightening during suturing, eliminating the need for knots, and facilitating a robust muscle-fascia layer approximation. This minimizes potential spaces that could lead to blood accumulation and infection. Furthermore, biomechanical studies have demonstrated that barbed sutures are less prone to failure under cyclical loading and exhibit greater dehiscence resistance than traditional braided sutures [32–34]. Also, some barbed sutures such as STRATAFIX™ are antibiotic-coated, which improves the antimicrobial properties of these barbed sutures and reduces the incidence of surgical site infections [35].

In Mun et al.‘s study [36], the authors observed that wound complications appeared later in patients treated with barbed sutures than in those treated with conventional sutures. This is likely due to the higher maintained tensile strength of barbed sutures. Despite this evidence, the data on wound complications between the two groups was insufficient for broad generalization especially in the presence of contradicting findings. For example, studies by Delgado-López et al. [25] and Chen et al. [8] reported 10 and 15 months follow up and reported comparable postoperative superficial wound infection and deep wound infection [25] and wound complications [8] between traditional suturing group and barbed suturing group. Notably, in Delgado-López et al. [25] study, some of cases required reoperation because of infection within the first month postoperatively and authors were able to confirm the internal stability of the tissue approximation when using barbed sutures. Hence, this meta-analysis was necessary to support the safety of barbed sutures in elective posterior surgery.

Lastly, it is essential to comment on the cost analysis performed by some authors. Although the costs of suture material were higher in the barbed sutures group (US$62.54 [30] or 20€ [25] average difference per patient), the total cost of closure time, including sutures, favored the barbed group [8, 25, 30, 37, 38]. Mansour et al. [30] showed that using barbed sutures will have a subsequent cost saving in hospital charges of US$884.60 per patient or US$617, as reported by Johnston et al. [37]. Chen et al. [8] also highlighted that the difference in barbed sutures cost was minimal compared to the total cost of the operation; however, their hospital did not charge for longer stay; hence complete cost analysis was conducted. Shi et al. [38] reported contradicting results as in the hospital carried out the study, longer operating room time did not increase hospitalization expenses; hence, the use of two sutures did not lead to significant differences in total hospitalization costs (¥103,654 for barbed sutures and ¥108,713 for conventional sutures).

## Strengths and limitations

This is the first meta-analysis comparing the clinical outcomes of barbed sutures and conventional sutures for wound closure in spine surgeries. We have statistically confirmed the reduction in suturing time and operating time based on evidence from multiple studies. However, there are several limitations. Firstly, only one RCT was included in the analysis, with most studies being retrospective. These studies are more susceptible to selection bias and confounding variables. We observed that three studies were of poor quality, which may limit the robustness and generalizability of the findings. Secondly, heterogeneity was observed in the primary outcome (operative time). However, we resolved the heterogeneity through sensitivity analysis, and the results remained consistent. Thirdly, due to the low number of studies, we could not perform subgroup analysis based on demographic characteristics, as only one study involved patients younger than 18. Fourthly, only three studies reported the follow-up (minimum 3 to 15 months), suggesting that more data is needed to validate the complications. Lastly, our analysis relied on pooled data from diverse studies, each with inherent limitations. These limitations are shared across the included studies.

## Conclusion

The barbed suture technique is deemed safe and effective for wound closure in spine surgeries. It has demonstrated superiority over the conventional suture technique by reducing the actual operation and suturing times. Both approaches yielded comparable outcomes regarding hospital stay duration, postoperative wound complications, infections, dehiscence or seroma, and hematoma or reintervention. It is recommended that clinical facilities conduct cost-analysis studies to ascertain the economic value of the barbed suture technique, which is primarily derived from its ability to decrease operation time.

## Electronic supplementary material

Below is the link to the electronic supplementary material.

Supplementary Material 1

## Data Availability

No datasets were generated or analysed during the current study.
