# Supplementary material for: Barbed sutures versus conventional sutures for wound closure in spine surgeries: a systematic review and meta-analysis
Source: Neurosurg Rev. 2024 Oct 10;47(1):769. doi: 10.1007/s10143-024-02909-9 (PMC11464557; doi:10.1007/s10143-024-02909-9)

**Supplementary Online Content**

**Barbed Sutures Versus Conventional Sutures for Wound Closure in Spine Surgeries: A Systematic Review and Meta-Analysis**

**eAppendix 1.** Sensitivity analysis for Primary and Secondary Outcomes

**eAppendix 2.** Funnel Plots Assessing Publication Bias for Primary Outcomes

**eAppendix 1.** Sensitivity analysis for Primary and Secondary Outcomes

A) Operative time


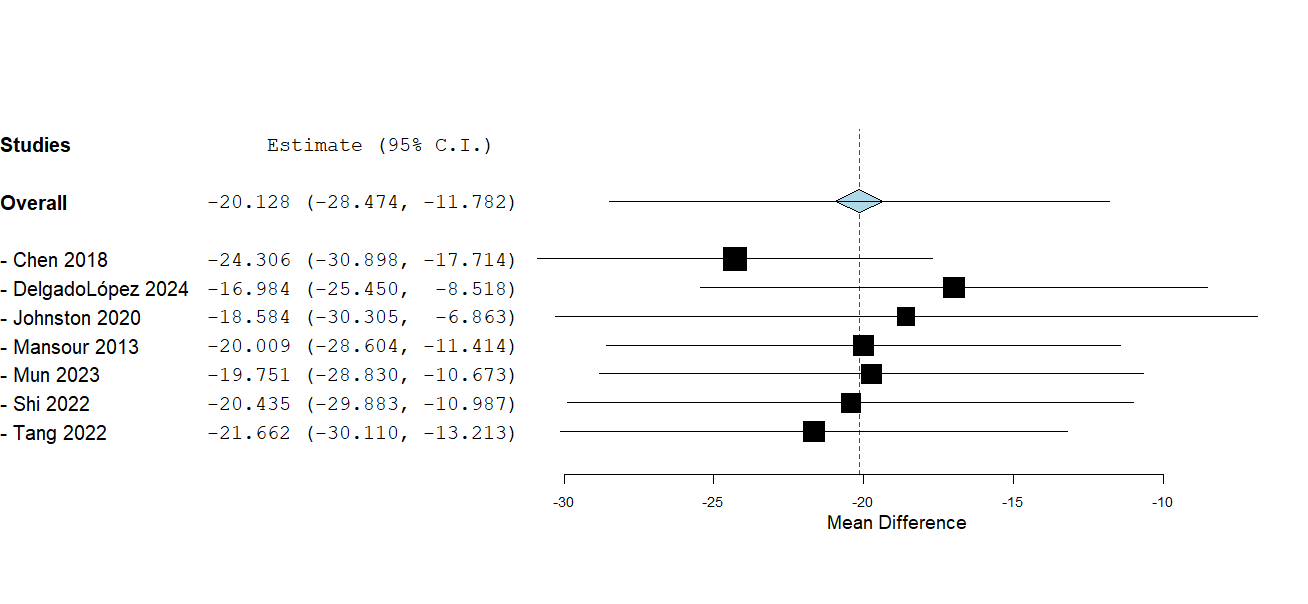


B) Wound closure time


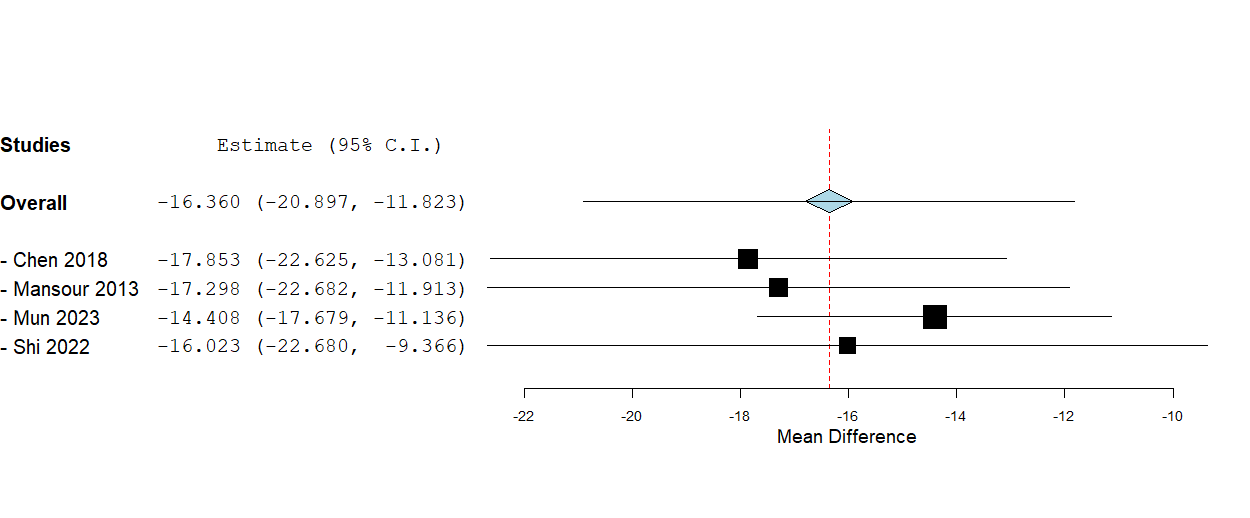


C) Post-operative wound complications


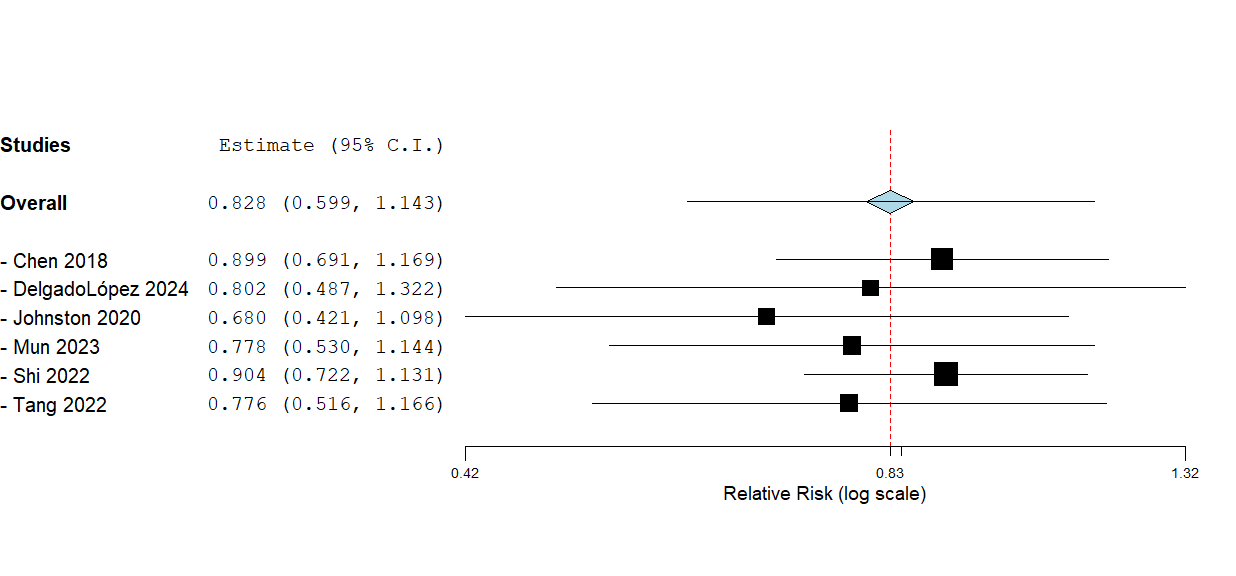


D) Post-operative infection


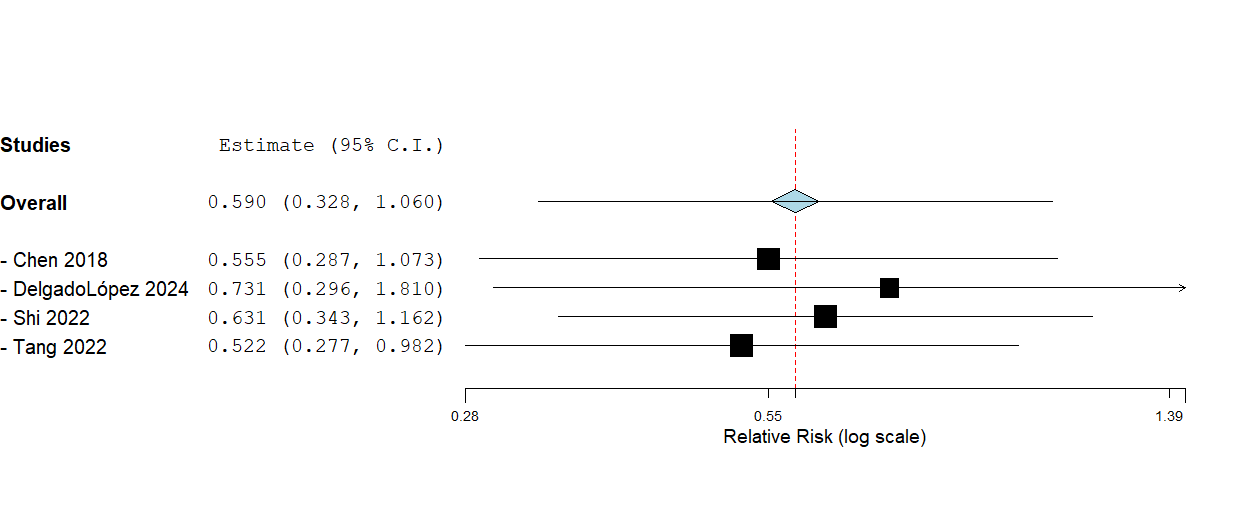


E) Wound dehiscence/seroma


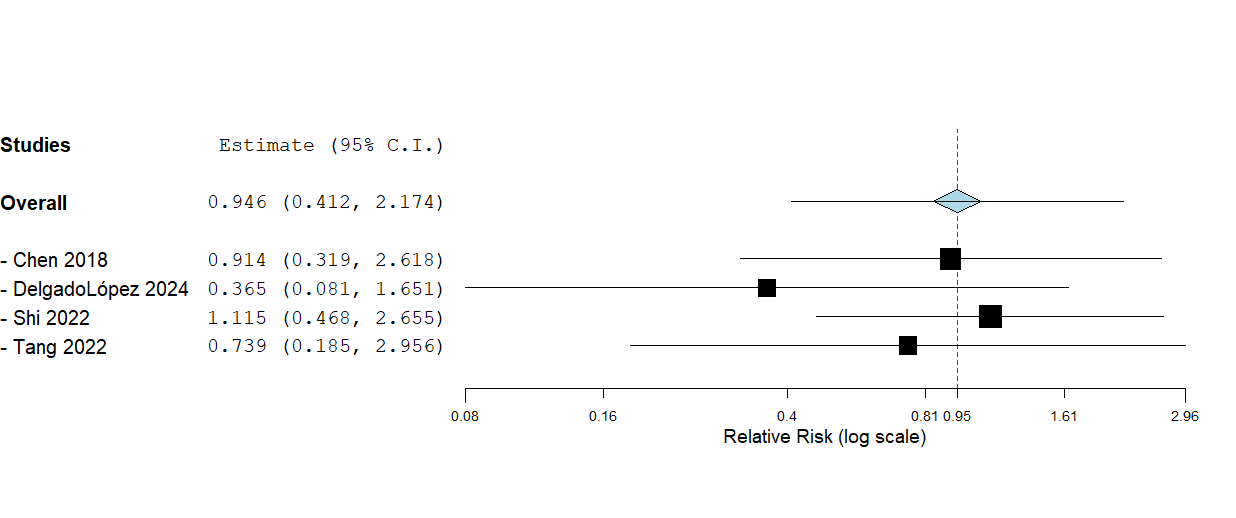


F) Length of hospital stay


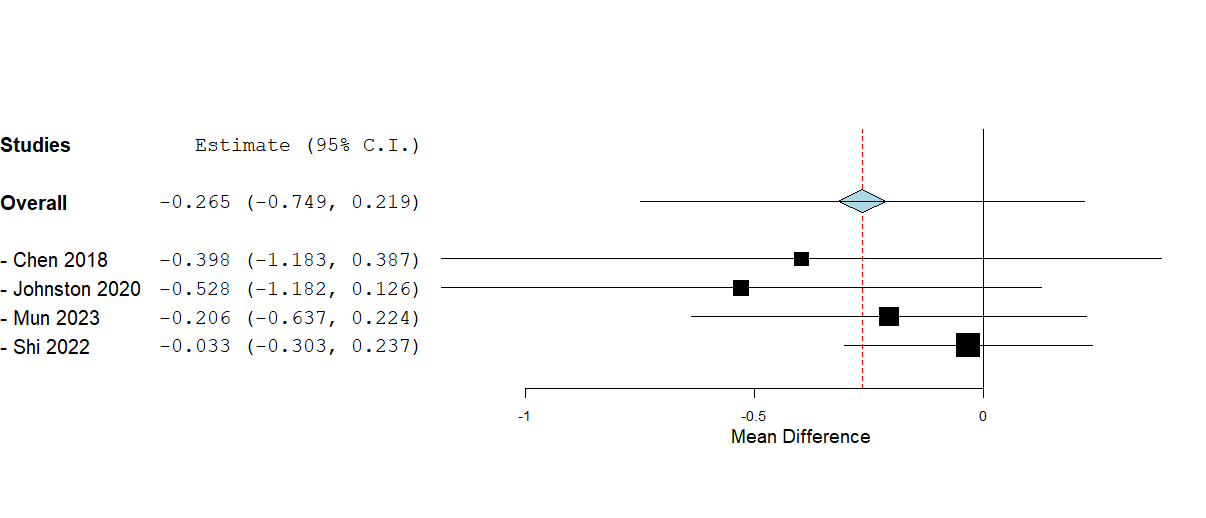


G) Reintervention


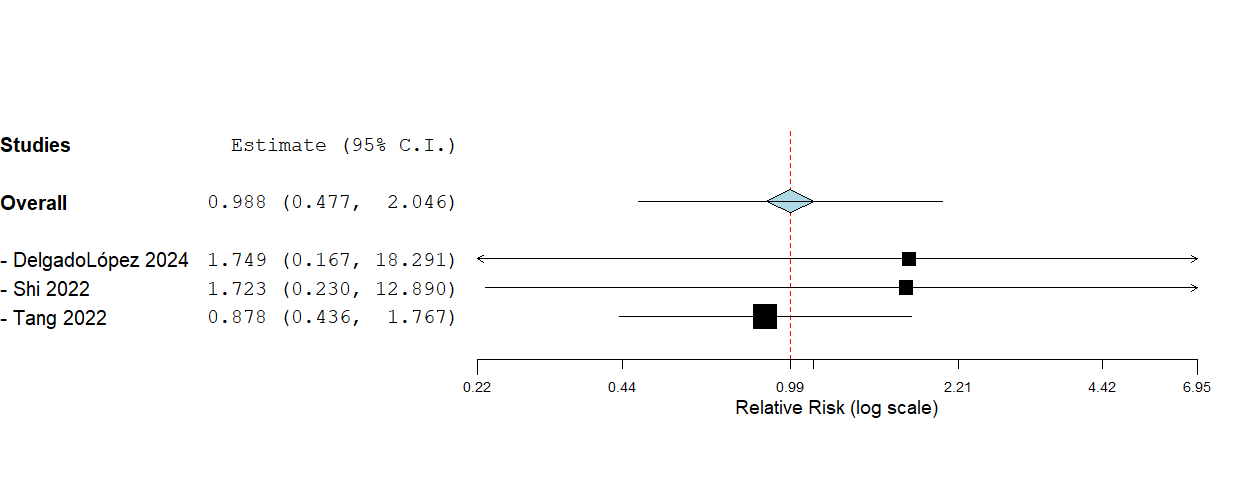


**eAppendix 2.** Funnel Plots Assessing Publication Bias for Primary Outcomes

A) Operative time


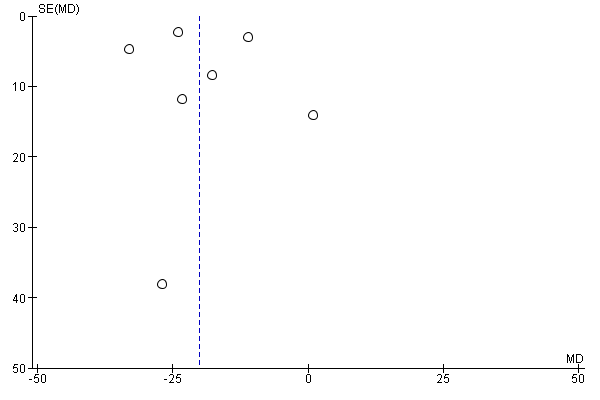


B) Wound closure time


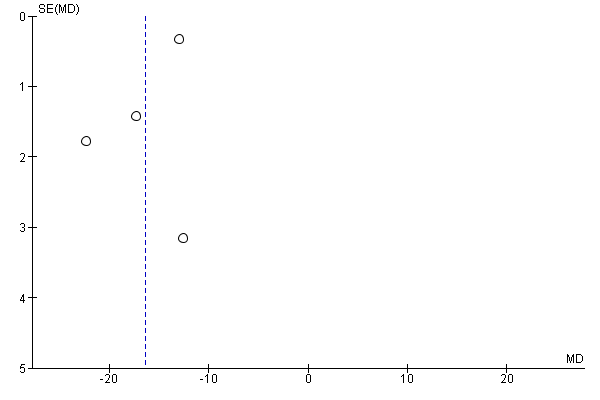


C) Post-operative wound complications


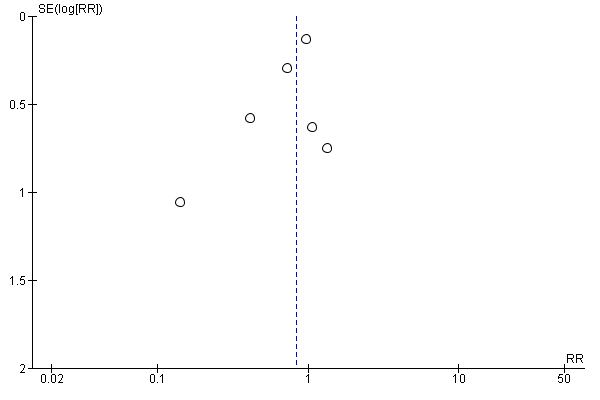

Supplement: Supplementary file 1 — Supplementary Material 1 [file 10143_2024_2909_MOESM1_ESM.docx]
